# Point-of-Care Ultrasound as a Key Diagnostic Tool in the Emergency Detection of Severe Pericardial Effusion

**DOI:** 10.7759/cureus.102253

**Published:** 2026-01-25

**Authors:** Diogo Dias Ramos, Amanda Hirschfeld, Inês Fiúza M. Rua, Mariana Alves Gaspar, Ana M Serrano

**Affiliations:** 1 Internal Medicine, Unidade Local de Saúde de São José, Lisbon, PRT; 2 Oncology, Instituto Português de Oncologia de Lisboa Francisco Gentil, Lisbon, PRT

**Keywords:** acute pericardial effusion, cardiac tamponade, dyspnea, emergency department, point-of-care ultrasound, point-of-care ultrasound (pocus)

## Abstract

Point-of-care ultrasound (POCUS) has become an essential extension of the physical examination in the emergency department, enabling rapid bedside assessment and timely clinical decision-making. We report a case of a 58-year-old female presenting with progressive dyspnea initially suggestive of a pulmonary etiology. Chest radiography revealed a right pleural effusion and apparent cardiomegaly. Bedside POCUS promptly identified a large circumferential pericardial effusion with echocardiographic signs of early cardiac tamponade, leading to immediate cardiology referral, intensive care admission, and pericardial drainage. This case highlights the pivotal role of POCUS in the early detection of life-threatening pericardial disease and its impact on clinical management and patient outcomes.

## Introduction

Point-of-care ultrasound (POCUS) has become an increasingly valuable tool in emergency medicine, functioning as an extension of the physical examination and allowing rapid bedside evaluation. Its use facilitates early identification of critical conditions, guides further diagnostic workup, and accelerates therapeutic decision-making [1,2].

Dyspnea, chest pain, and shock are conditions susceptible to evaluation with ultrasound, considering diagnostic accuracy and clinical impact already proven. In patients presenting with dyspnea, POCUS is particularly useful in distinguishing between cardiac, pulmonary, and pleural causes and demonstrates near-perfect concordance with final diagnoses in patients presenting to the emergency department with acutely decompensated heart failure, acute coronary syndromes, and shock.

Moreover, growing evidence indicates that its use is associated with reduced hospital length of stay, lower mortality, and improved efficiency and cost-effectiveness of care, largely through earlier diagnosis, expedited clinical decision-making, and more targeted therapeutic interventions [3-5].

We present a case in which POCUS was crucial for the timely diagnosis of a severe pericardial effusion, significantly altering clinical management and improving patient outcome.

## Case presentation

A 58-year-old female presented to the emergency department with progressive dyspnea on minimal exertion. Her medical history included arterial hypertension, dyslipidemia, obesity, cutaneous lupus erythematosus, and bronchial asthma. She was an active smoker. The patient reported having received vaccination against SARS-CoV-2 in February 2022, after which she developed intermittent dyspnea requiring short-acting bronchodilator use approximately twice weekly.

Her regular medication included salbutamol as needed, perindopril 5 mg once daily, rosuvastatin 10 mg once daily, and acetylsalicylic acid 100 mg once daily. She denied chest pain, fever, cough, palpitations, or syncope.

On examination, the patient was tachypneic (respiratory rate of 26 breaths/min), with an oxygen saturation of 93-94% on room air, blood pressure of 132/78 mmHg, heart rate of 104 beats/min, and body temperature of 36.8°C. Pulmonary auscultation revealed diffuse wheezing, and mild bilateral peripheral edema was noted. Heart sounds were audible without obvious muffling.

Laboratory evaluation was unremarkable, including normal cardiac biomarkers, with no clinically significant rise on the second determination (Table 1).

**Table 1:** Laboratory evaluation.

Blood work	Patient results	Normal range
Hemoglobin	15.0 g/dL	12.0 – 15.0 g/dL
Hematocrit	46.8%	35 – 46%
Mean corpuscular volume (MCV)	95.5 fL	78.0 – 96.0 fL
Mean corpuscular hemoglobin (MCH)	30.6 pg	26.0 – 33.0 pg
Leukocytes	8.95 ×10⁹/L	4.5 – 11.0 ×10⁹/L
Neutrophils (absolute count)	6.15 ×10⁹/L	2.0 – 8.5 ×10⁹/L
Eosinophils (absolute)	0.12 ×10⁹/L	0.0 – 0.6 ×10⁹/L
Basophils (absolute)	0.06 ×10⁹/L	0.0 – 0.1 ×10⁹/L
Lymphocytes (absolute)	1.77 ×10⁹/L	0.9 – 3.5 ×10⁹/L
Monocytes (absolute)	0.85 ×10⁹/L	0.2 – 1.0 ×10⁹/L
Platelets	256 ×10⁹/L	150 – 450 ×10⁹/L
Glucose	103 mg/dL	60 – 100 mg/dL
Urea	29 mg/dL	21.0 – 43.0 mg/dL
Creatinine	0.78 mg/dL	0.57 – 1.11 mg/dL
Sodium	142 mmol/L	136 – 145 mmol/L
Potassium	5.0 mmol/L	3.5 – 5.1 mmol/L
Chloride	101 mmol/L	98 – 107 mmol/L
C-reactive protein (CRP)	16.5 mg/L	<5.0 mg/L
High-sensitivity troponin I	20.4 pg/mL	<15.6 pg/mL
Myoglobin	59.8 ng/mL	<106.0 ng/mL
D-dimer	539 µg/L	<230 µg/L
High-sensitivity troponin I (repeat)	22.6 pg/mL	<15.6 pg/mL
N-terminal pro-B-type natriuretic peptide (NT-pro-BNP)	274 pg/mL	<352 pg/mL

**Figure 1:**
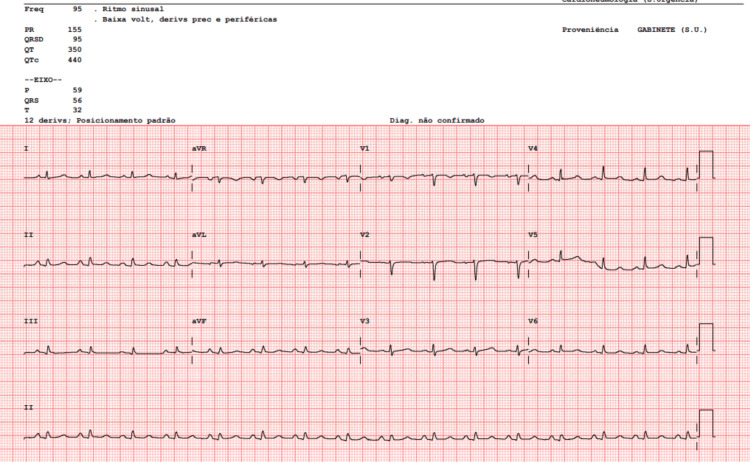
Electrocardiography showing sinus rhythm at 95 beats per minute, low-voltage QRS complexes, and T-wave inversion in leads V1 and aVR.

Electrocardiography demonstrated sinus rhythm at 95 beats per minute, low-voltage QRS complexes, and T-wave inversion in leads V1 and aVR (Figure 1).

**Figure 2:**
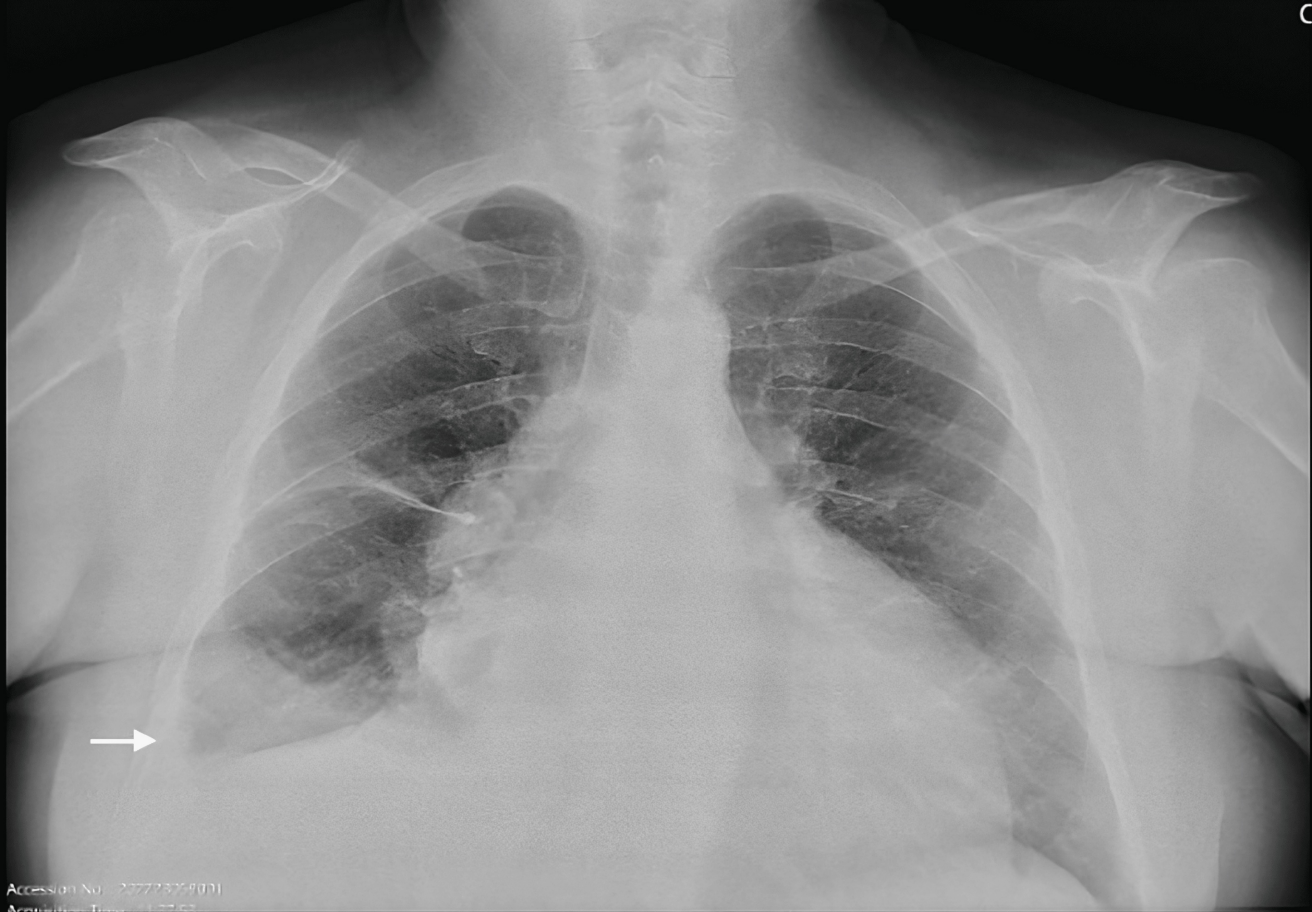
Chest radiography showing a small right-sided pleural effusion with fissural thickening and enlargement of the cardiac silhouette.

Chest radiography revealed a right-sided pleural effusion with fissural thickening and apparent enlargement of the cardiac silhouette (Figure 2).

**Figure 3:**
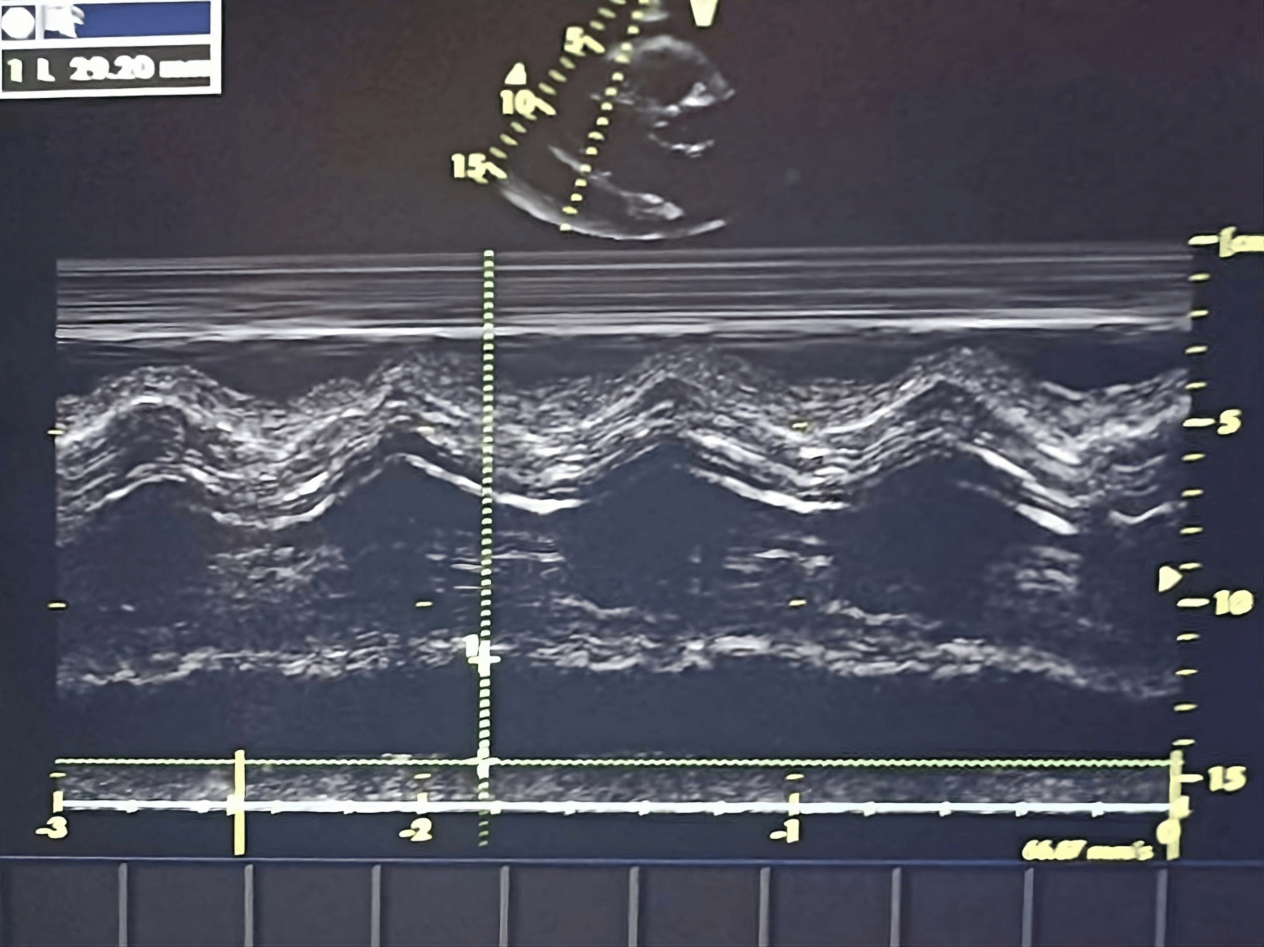
Bedside cardiac ultrasound showing a 29.20 mm posterior pericardial effusion.

Given the discrepancy between clinical findings and radiographic cardiomegaly, bedside POCUS was performed. Cardiac ultrasound demonstrated a large circumferential pericardial effusion (Video 1), measuring up to 29 mm posteriorly, with a swinging heart and partial right ventricular diastolic collapse, consistent with impending cardiac tamponade (Figure 3).

**Video 1:** Bedside cardiac ultrasound showing a large circumferential pericardial effusion.

Cardiology was immediately consulted, and the patient was transferred to the cardiac intensive care unit for further evaluation and management. Transthoracic echocardiography confirmed a severe pericardial effusion (23 mm anteriorly), compression of right-sided cardiac chambers, and a dilated inferior vena cava (23 mm) with reduced inspiratory collapse. Left ventricular systolic function was preserved, and no hemodynamically significant valvular abnormalities were identified.

A diagnosis of acute pericarditis with severe pericardial effusion was established. Treatment with nonsteroidal anti-inflammatory drugs and colchicine was initiated. Pericardiocentesis was performed with placement of a pericardial drain, which remained in situ for 48 hours without complications. Cytological analysis and microbiological cultures, including aerobic, anaerobic, and mycobacterial studies, were negative. The patient showed marked clinical improvement and recovered uneventfully.

## Discussion

This case highlights the clinical value of POCUS in the emergency department for the rapid identification of life-threatening cardiac conditions. Acute dyspnea is a frequent presenting complaint and encompasses a broad and heterogeneous differential diagnosis, including pulmonary, cardiac, vascular/thromboembolic, metabolic, and infectious causes. Although it is often initially attributed to pulmonary pathology, particularly in patients with known respiratory comorbidities, critical cardiac conditions such as acute decompensated heart failure, acute coronary syndromes, pericardial tamponade, and massive pulmonary embolism must be promptly considered and excluded, given their associated morbidity and mortality [6,7]. In this context, POCUS has emerged as an increasingly sensitive, rapid, and user-friendly bedside tool, enabling early differentiation among these etiologies and facilitating timely, targeted clinical management [3-6].

Physical examination and chest radiography have limited sensitivity for early detection of pericardial effusion. Classical signs of tamponade (right atrial or right ventricular diastolic collapse, plethoric inferior vena cava, and respiratory variation in ventricular filling) are frequently absent until late in the disease course, and chest radiographs may only demonstrate cardiomegaly in large or chronic effusions. Current European Society of Cardiology guidelines recommend transthoracic echocardiography as the first-line diagnostic modality in suspected pericardial disease, given its ability to rapidly confirm pericardial effusion and evaluate its hemodynamic impact [8].

In this patient, POCUS enabled immediate bedside diagnosis of a severe pericardial effusion with echocardiographic signs of hemodynamic compromise, prompting urgent cardiology consultation and timely intervention. Echocardiographically assisted diagnosis has been shown to allow identification of tamponade physiology before the development of overt hypotension or shock, facilitating earlier intervention and preventing further clinical deterioration [9,10].

Beyond its diagnostic role, POCUS significantly influences clinical decision-making in the emergency setting. In patients presenting with acute dyspnea, ultrasound-based assessment improves diagnostic accuracy and shortens time to appropriate treatment when compared with standard evaluation alone [11]. Additionally, early bedside echocardiography may reduce the need for further diagnostic investigations, such as computed tomography, which are more time-consuming and may delay definitive management in unstable patients [12].

Despite its advantages, POCUS remains an operator-dependent modality, requiring adequate training, experience, and continuous quality assurance to ensure diagnostic accuracy. Moreover, access to POCUS differs markedly between resource-rich and resource-limited settings, raising important concerns regarding healthcare equity. Recent evidence underscores that disparities in access to ultrasound technology and training may contribute to inequities in diagnostic capability and clinical outcomes, particularly in underserved populations. Addressing these gaps through affordable technology, standardized education, and global training initiatives is essential to promote the equitable integration of POCUS into emergency care [9,12,13].

## Conclusions

POCUS is a powerful diagnostic modality in the emergency department that extends beyond conventional imaging findings, such as an enlarged cardiac silhouette on chest radiography. In this case, its use allowed early recognition of a severe pericardial effusion and facilitated prompt, potentially life-saving intervention. Notably, this presentation was particularly challenging due to the nonspecific nature of the initial symptoms and the predominance of respiratory complaints, which could have easily led to diagnostic anchoring on a primary pulmonary etiology. The absence of classical signs of cardiac tamponade and the initial hemodynamic stability further increased diagnostic complexity.

This case, therefore, underscores the unique value of early bedside ultrasound in uncovering occult, life-threatening cardiac pathology in patients with undifferentiated dyspnea, highlighting its pivotal role in preventing diagnostic delay and adverse outcomes. Routine integration of POCUS into emergency care and continued investment in clinician training are essential to maximize its clinical impact.
